# ASSOCIATION OF SUBJECTIVE AND OBJECTIVE COGNITION WITH DISEASE AWARENESS: EVIDENCE FROM BLOOD BIOMARKERS DATA

**DOI:** 10.1093/geroni/igac059.2262

**Published:** 2022-12-20

**Authors:** Zhuoer Lin, Mingqi Fu, Xi Chen

**Affiliations:** Yale University, New Haven, Connecticut, United States; Wuhan University, Wuhan, Hubei, China (People's Republic); Yale University, New Haven, Connecticut, United States

## Abstract

Impaired cognitive ability and its misperception contribute to poor decision-making of older adults; yet their impact on health-related decisions is less known. We examined how self-perceived and actual cognition were associated with chronic disease awareness using a nationally representative sample of Chinese older adults. Blood biomarkers data were collected in 2015 to identify participants’ dyslipidemia and diabetes status. Among participants with identified dyslipidemia or diabetes, disease awareness was defined as self-reported diagnosis of the conditions as of 2018. Objective and subjective cognition were respectively assessed using the Mini-Mental State Examination and self-rated memory. The associations of subjective and objective cognition with chronic disease awareness were determined by weighted logistic regressions. Among 4,578 adults aged 60 and older with complete measurements, 1,442 and 759 individuals were identified having dyslipidemia and diabetes, with proportions of disease awareness being 38.0% and 58.1%, respectively. Individuals with mild (Odds Ratio [OR]=0.63; 95%CI: 0.45-0.89) or severe cognitive impairment (OR=0.46; 95%CI: 0.29-0.72) had lower odds of dyslipidemia awareness; and those with severe cognitive impairment had lower odds of diabetes awareness (OR=0.43; 95%CI: 0.23-0.79) than cognitively intact counterparts. However, adjusting for objective cognition, older adults with better subjective cognition had lower odds of dyslipidemia (OR=0.80; 95%CI: 0.63-1.02) and diabetes (OR=0.71; 95%CI: 0.55-0.92) awareness. Such associations were stronger for individuals with rural status, lower education, or living without children. Our findings highlight the great challenges deteriorated cognitive function and misperception may pose to chronic disease awareness, as well as the importance of targeted supports particularly for those more disadvantaged.

